# Effects of plyometric training on explosive power and swimming performance in adolescent swimmers: a systematic review and meta-analysis with dose-response analysis

**DOI:** 10.1186/s13102-026-01989-y

**Published:** 2026-08-14

**Authors:** Rangxi Jin, Mitchell James Finlay, Francisco Cuenca-Fernández, Wei Huang

**Affiliations:** 1 School of Athletic Performance, Shanghai University of Sport, Shanghai, 200438 China; 2 Sport Department, University Academy 92, Manchester, UK; 3 Department of Sports and Computer Sciences, Universidad Pablo de Olavide, Seville, Spain; 4 Department of Physical Education and Sports, Faculty of Sport Sciences, Aquatics Lab, University of Granada, Ctra. Alfacar SN, Granada, 18071 Spain

**Keywords:** Plyometric training, Adolescent swimmers, Explosive power, Dose-response relationship, Systematic review and meta-analysis

## Abstract

Adolescent swimmers depend heavily on lower-limb power during critical phases such as the block start, wall turn, and sprints, with the circa-peak height velocity (PHV) period representing a sensitive window for neuromuscular development. Plyometric training (PT), utilizing the stretch-shortening cycle, is a key component of long-term athletic development, yet evidence stratified by maturation stage is lacking. This systematic review and meta-analysis searched PubMed, Web of Science, Embase, EBSCO, Cochrane Library, and CNKI for controlled trials in swimmers aged 10–18 years, following PRISMA 2020. Effect sizes were pooled using Hedges’ g in a random-effects model, dose-response relationships were evaluated via meta-regression, methodological quality assessed with RoB 2.0 and PEDro, and evidence certainty rated by GRADE. Findings suggest that PT may be associated with moderate-to-large positive point estimates for dry-land explosive power and short-distance swimming performance (with positive effect sizes oriented so that faster swim times yield positive g), with point estimates that appeared larger around the circa-PHV stage; however, none of the pooled estimates reached statistical significance under the primary Hartung–Knapp analysis, the confidence intervals were wide, and all findings should therefore be read as preliminary and hypothesis-generating rather than as confirmation that PT significantly improves these outcomes. Transfer to longer distances was smaller in magnitude. The results provide a provisional basis for tailoring PT programmes by developmental stage that requires confirmation in larger studies.

**Supplementary Information:**

The online version contains supplementary material available at https://doi.org/10.1186/s13102-026-01989-y.

## Introduction

Performance in the key technical phases of the block start, wall turn and in-water acceleration in adolescent swimmers is heavily dependent on instantaneous power output released by the lower limbs and trunk [1]. Within sprint swimming events, upper- and lower-body maximum strength levels show a robust positive association with competition performance, and lower-limb explosive power contributes particularly prominently to push-off from the starting block and to ground reaction output from the pool wall [2], while the start and turn phases together can account for nearly one third of total race time and map directly onto final competitive standing [3]. The circa-peak height velocity (PHV) period of adolescence is regarded as the most sensitive window for neuromuscular plasticity, during which increases in tendon stiffness, maturation of motor unit recruitment and improvements in neural drive efficiency jointly constitute the physiological basis for the rapid development of explosive power [4], and the application of structured explosive training stimuli during this stage has been shown to be both safe and capable of eliciting substantially amplified training responses, thereby establishing the biological rationale for explosive-power-oriented dry-land interventions in adolescent swimming populations [5].

Plyometric training (PT) enhances the elastic energy utilisation of the musculotendinous complex and the rate of neural drive through the rapid stretch-shortening cycle (SSC) [6], and occupies a central position within the long-term athletic development framework for young athletes as a primary bridging modality between fundamental motor skill and sport-specific strength [7]. Large-sample evidence from track-and-field and team-sport settings has confirmed the significant improving effects of PT on jumping, sprinting and change-of-direction performance [8], and comprehensive evaluation in water sports has further indicated that while dry-land explosive power improvements are clearly demonstrable, the magnitude of transfer to in-water time performance remains comparatively limited [8]. Existing reviews targeting competitive swimmers have generally pooled heterogeneous training modalities and competition distances without stratifying by developmental stage [9], which has made precise attribution of effect differences across intervention modes difficult to achieve [10], and systematic reviews of the relationship between dry-land resistance training and start performance likewise present methodological limitations in sample heterogeneity and incomplete dose reporting [11]. The qualitative evidence accumulated to date has yet to clearly answer three core questions regarding for whom PT is effective, at what dose it is effective, and for which swimming outcomes it is effective, and the quantitative integration of available evidence therefore stands out as an urgent research gap to be filled.

Adolescent swimmers aged 10 to 18 years span multiple biological maturation stages, and the integrated effect of dry-land PT on explosive power and sport-specific performance in this population has not been subject to quantitative evidence synthesis. Randomised controlled trials in prepubertal male swimmers have shown that 8 weeks of PT can produce moderate to large effects on countermovement jump (CMJ) and standing long jump (SLJ) performance and can improve 25 m front-crawl time [12], while comparable protocols in prepubertal female swimmers demonstrate concurrent improvements in explosive power and sport-specific performance, yet quantitative cross-study comparisons of sex- and maturation-related differential responses remain lacking [13]. The three core innovations of the present work reside in conducting a systematic review and meta-analysis specifically for the 10-to-18-year developmental stage population, in quantifying the dose-response relationship between effect size and two core dose variables, namely intervention weeks and total ground contacts, through meta-regression, and in evaluating comprehensively the gradient of improvement across dry-land explosive power and swimming performance over different distances. Three research hypotheses are advanced on this basis, with H1 stating that PT produces effects of at least moderate magnitude on both explosive power and swimming performance in adolescent swimmers, H2 proposing that a positive gradient exists between intervention weeks and effect magnitude with an identifiable dose window of stable effect, and H3 anticipating that the effect on dry-land explosive power exceeds that on in-water performance and is modulated by maturation status. The present study aims to provide an evidence base for dry-land training prescription in adolescent swimming and to offer coaches an actionable quantitative reference for individualising PT programmes according to developmental stage.

## Methods

### Protocol registration, search strategy and eligibility criteria

The study was prospectively registered in the PROSPERO international prospective register of systematic reviews, and the entire process adhered strictly to the Preferred Reporting Items for Systematic Reviews and Meta-Analyses (PRISMA) 2020 reporting guideline to safeguard the transparency and reproducibility of the search, screening, assessment and synthesis stages [14], PROSPERO: CRD420251127520, registered January 2026. Given that the present work constitutes a secondary systematic synthesis of already published literature and did not directly recruit human participants or collect primary data, separate approval from an institutional ethics review board was not required, and the ethical compliance of each included original study had been approved by the respective research teams at their publishing institutions and declared within the corresponding original papers. The systematic search covered six databases, namely PubMed, Web of Science, Embase, EBSCO Academic Search Complete, Cochrane Library (CENTRAL) and CNKI, with a cut-off date set at April 2026, and the search strategy was built around core subject headings and free text terms including “plyometric training”, “jump training”, “stretch-shortening cycle”, “adolescent”, “swimmer” and “swimming performance”, combined through Boolean logic in accordance with the controlled vocabulary of each database, supplemented by backward citation tracking of included studies and grey-literature searching through Google Scholar to minimise the risk of omission. The full database-specific search strings, the number of records identified per source and the supplementary search steps are reported verbatim in Supplementary File S1, and the reports sought but not retrieved within the search window together with the records excluded at title-and-abstract screening and their reason categories are listed in Table S1. The Population, Intervention, Comparator, Outcome and Study design (PICOS) framework was operationalised as follows, with the Population (P) defined as healthy swimmers aged 10 to 18 years, the Intervention (I) defined as structured PT of no less than 4 weeks with extractable programme parameters, the Comparator (C) defined as routine swimming training or any non-PT programme, the Outcome (O) defined as indicators of explosive power or sport-specific swimming performance, and the Study design (S) comprising randomised controlled trials (RCT) and non-randomised controlled trials. Key exclusion criteria encompassed studies in which PT was combined with additional newly introduced training without a means to isolate independent effects, cross-sectional designs reporting only acute effects, and reports failing to provide extractable means and standard deviations, in order to safeguard the statistical validity of effect size pooling.

### Data extraction, methodological quality and certainty of evidence

Data extraction was conducted independently by two reviewers, with extracted variables encompassing sample size, age, sex composition, biological maturation (characterised through maturity offset or Tanner stage), training history, PT programme parameters (including intervention weeks, weekly frequency, ground contacts per session, intensity grading and whether progressive overload was applied), control condition settings, as well as pre- and post-intervention outcome means and standard deviations. The methodological risk of bias was assessed in parallel using the Cochrane Risk of Bias tool version 2.0 (RoB 2.0) and the Physiotherapy Evidence Database (PEDro) scale, with the former focusing on the five domains of randomisation process, deviation from intended intervention, missing outcome data, outcome measurement and selective reporting [15], and the latter providing complementary scoring of external and internal validity through its 10 items, so that their parallel application simultaneously accommodates the dual requirements of structured bias judgement and quantitative quality scoring. The certainty of evidence was rated through the Grading of Recommendations, Assessment, Development and Evaluation (GRADE) system, which integrates considerations of study limitations, inconsistency, indirectness, imprecision, publication bias, effect magnitude and dose-response relationships to classify the certainty of evidence into four levels of high, moderate, low and very low [16]. When original studies did not report Tanner staging or maturity offset explicitly, maturation status was inferred from chronological age, and studies with mixed-sex samples spanning early- to late-pubertal ages that could not be unambiguously classified into a single PHV category were retained for effect size pooling but labelled separately and excluded from PHV-based subgroup analysis. Any disagreements during data extraction or quality assessment were arbitrated by a third senior reviewer to ensure objectivity and consistency of judgement.

### Effect size pooling, dose-response modelling and heterogeneity/bias testing

Effect sizes were expressed as small-sample-bias-corrected Hedges’ *g* For swimming-time outcomes the sign was oriented by taking the control-minus-intervention contrast, so that a positive g denotes faster performance, that is a shorter race time, and for jump and power outcomes a positive g denotes a higher measured value, and the pooling model adopted the restricted maximum likelihood (REML) approach for estimation of the between-study variance, given that the restricted maximum likelihood estimator exhibits smaller downward bias than the DerSimonian–Laird estimator when the number of studies is small, and inference on each pooled estimate relied on the Hartung–Knapp–Sidik–Jonkman adjustment with a t reference distribution on k − 1 degrees of freedom, incorporating the max(q, 1) safeguard to avoid anti-conservative intervals, whereas the DerSimonian–Laird random-effects model with a normal reference was retained as a sensitivity analysis reported in Table S2 to accommodate true between-study heterogeneity, with statistical heterogeneity quantified jointly through the *I*^*2*^ index and the *τ*^2^ estimate, and *I*^*2*^ values exceeding 50% interpreted as indicative of substantial heterogeneity. Prespecified moderators were centred on biological maturation (Pre-PHV, Circa-PHV and Post-PHV), supplemented by sex, training level and intervention mode (add-on versus replacement) as three additional dimensions for subgroup comparison. PHV-based subgroup analysis was restricted to studies with a clearly identified Pre-PHV or Circa-PHV classification, whereas studies without unambiguous PHV categorisation and the single Post-PHV study were excluded from formal subgroup comparisons to maintain analytic rigor. Given that the included studies covered only two frequency levels of 2 and 3 sessions per week and that intensity reporting exhibited considerable heterogeneity, the originally planned four-dimensional restricted cubic spline model could not be stably identified at the level of implementation and was accordingly downgraded to univariable linear meta-regression, which focused on testing the linear trend between effect size and the two core dose variables of intervention weeks and total ground contacts in order to derive an interpretable dose-response relationship. Sources of heterogeneity were explored through leave-one-out sensitivity analysis performed by successively omitting individual studies to probe the robustness of the pooled results. Formal assessment of publication bias was not conducted given that the final number of included studies k equalled 8 and fell below the recommended threshold of k ≥ 10 for funnel plot inspection and Egger testing, thereby avoiding misinterpretation arising from low statistical power. For any outcome informed by only two studies the Hartung–Knapp–Sidik–Jonkman interval reduces to a t distribution on a single degree of freedom and yields a confidence interval too wide to support interpretation, so such outcomes were summarised through their pooled point estimate and the direction and magnitude of the contributing studies rather than through inferential testing. All analyses were carried out in the R 4.6.0 environment using the metafor 5.0.1 and meta 8.5.0 packages, with statistical significance set at *P* less than 0.05.

## Results

### Study selection process and baseline characteristics of included studies

**Fig. 1:**
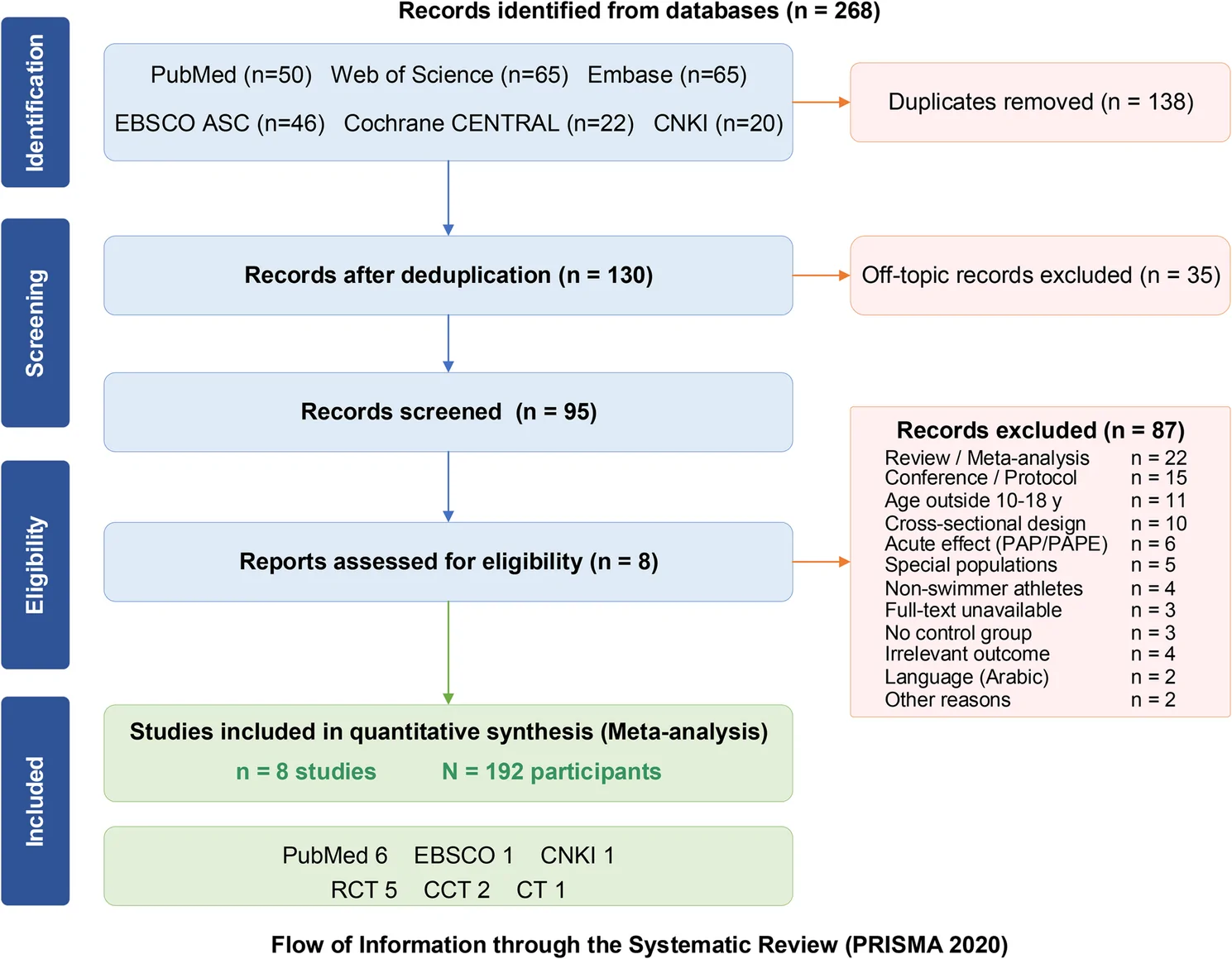
PRISMA 2020 flow diagram of the study selection process

The systematic search across the six databases identified a total of 268 records (PubMed 50, Web of Science 65, Embase 65, EBSCO 46, Cochrane CENTRAL 22 and CNKI 20). After deduplication, 130 records were retained, and following subject-level screening that excluded 35 clearly off-topic records, 95 records entered the title/abstract and full-text screening phase, at which point a cumulative total of 87 records were excluded for reasons that included systematic reviews and meta-analyses (*n* = 22), conference abstracts and registered protocols (*n* = 15), age outside the 10-18-year range (*n* = 11), cross-sectional or observational designs (*n* = 10), acute effect studies (*n* = 6), studies in special populations (*n* = 5), outcome indicators irrelevant to the present research question (*n* = 4), samples not consisting of swimmer athletes (*n* = 4), absence of a control group (*n* = 3), unavailable full text (*n* = 3), language restriction (*n* = 2) and other reasons (*n* = 2). The complete screening workflow is detailed in Fig. 1.

**Fig. 2:**
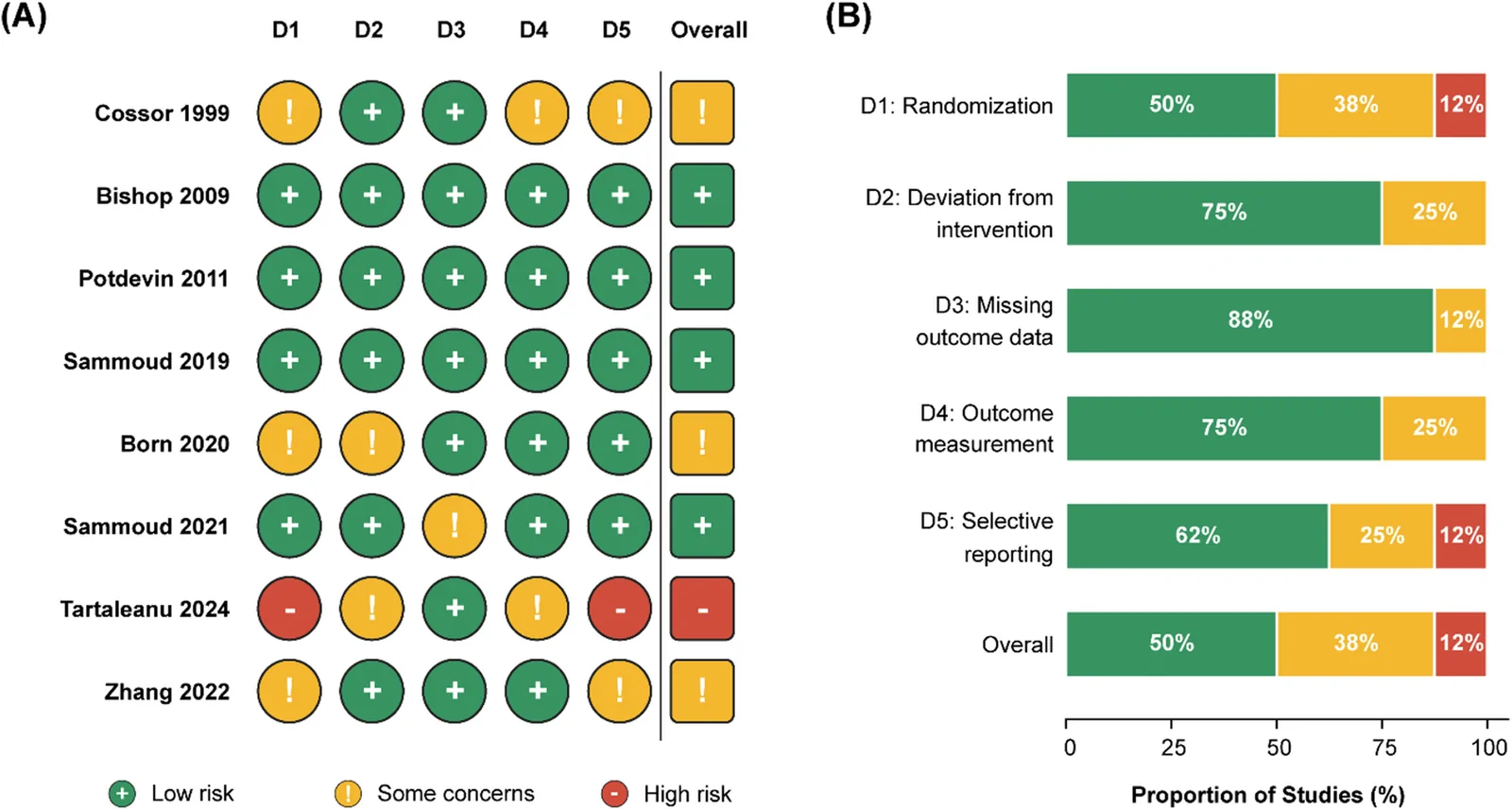
Methodological quality assessment of included studies (Cochrane RoB 2.0 traffic light plot). Methodological quality assessment of included studies using the Cochrane RoB 2.0 tool. **A** Risk-of-bias judgements for individual studies across the five RoB 2.0 domains (D1–D5) and overall bias assessment; **B** Proportional distribution of risk-of-bias classifications across the included studies.

Eight studies were ultimately included (5 RCTs, 2 controlled clinical trials (CCT) and 1 controlled trial (CT)), comprising a pooled sample of 192 adolescent swimmers aged 10.0 to 17.0 years, with mixed-sex composition in 4 studies (50%), males in 2 studies (25%) and females in 2 studies (25%), and maturation distribution of Pre-PHV in 2 studies, Circa-PHV in 4 studies, Post-PHV in 1 study and an early-puberty mixed stage in 1 study (Cossor 1999, mixed-sex sample aged 10–14 years that could not be unambiguously classified into a single PHV category). The PT programme parameters spanned intervention durations of 6 to 20 weeks, frequencies of 2 to 3 sessions per week and total ground contacts of 680 to 5400, while control conditions comprised replacement modes (*n* = 4, including full-swim replacement in 1 study, warm-up replacement in 2 studies and dry-land replacement in 1 study), add-on modes (*n* = 3 in [17–19] and an active maximal-strength-training (MST) comparator (*n* = 1 in [20]). The methodological quality assessment is presented in Fig. 2.

Figure 2(A) presents the ratings of each of the 8 studies across the 5 RoB 2.0 bias domains (D1-D5) and the overall judgement, while Fig. 2(B) summarises the proportional distribution of each bias domain across the included studies. Overall, 4 studies (50%) were rated as low risk, 3 studies (38%) as some concerns and 1 study (12%) as high risk, with a PEDro median score of 6.5/10 (interquartile range 5–7). Complete characteristics of the included studies are presented in Table 1.

**Table 1:** Characteristics of included studies

Study	Country	Design	*N* (PJT/Ctrl)	Age / Sex	Maturation	Duration (wks × freq)	Contacts	Intervention mode	Outcomes	RoB 2.0	PEDro
(Cossor et al., 1999) [21]	Australia	CCT	19/19	10–14 y / Mixed	Early-puberty	20 × 3/wk	~ 5400	Replace swim	50 m FC	Some concerns	5/10
(Bishop et al., 2009) [18]	Australia	RCT	11/11	13.6 y / Mixed	Circa-PHV	8 × 2/wk	NR	Add-on	Start velocity, Start distance, 5.5 m time	Low risk	7/10
(Potdevin et al., 2011) [19]	France	RCT	12/11	14.0 y / Mixed	Circa-PHV	6 × 2/wk	2146	Add-on	CMJ, SJ, 50 m FC, 400 m FC	Low risk	7/10
(Sammoud et al., 2019) [12]	Tunisia	RCT	14/12	10.2 y / Male	Pre-PHV	8 × 2/wk	680	Replace warmup	CMJ, SLJ, 15 m/25m/50m FC, 25 m WP/KWP	Low risk	7/10
(Born et al., 2020) [20]	Switzerland	CT	11/10	17.0 y / Mixed	Post-PHV	6 × 2/wk	NR	Add-on (vs. MST)	5 m, 15 m FC	Some concerns	5/10
(Sammoud et al., 2021) [13]	Tunisia	RCT	12/10	12.1 y / Female	Pre-PHV	8 × 2/wk	680	Replace warmup	CMJ, SLJ, 25 m/50m FC, 25 m WP/KWP	Low risk	7/10
(Țărțăleanu & Orțănescu, 2024) [17]	Romania	CCT	10/10	13–14 y / Male	Circa-PHV	6 × 2/wk	~ 960	Add-on	Reaction time, Start distance	High risk	3/10
(Zhang, 2022) [22]	China	RCT	10/10	12–14 y / Female	Circa-PHV	10 × 3/wk	~ 1050	Replace dryland	CMJ, SLJ, 10 m/25m/50m FC, 25 m Pull/Kick	Some concerns	6/10

*CCT* controlled clinical trial, *CMJ* countermovement jump, *CT* controlled trial, *FC* front crawl, *KWP* kick without push, *MST* maximal strength training, *NR* not reported, *PEDro* Physiotherapy Evidence Database scale, *PHV* peak height velocity, *PJT* plyometric jump training, *RCT* randomised controlled trial, *RoB* *2.0* Cochrane Risk of Bias tool version 2.0, *SJ* squat jump, *SLJ* standing long jump, *WP* with push

### Meta-analytic findings for explosive power: CMJ as the gold-standard anchor

**Fig. 3:**
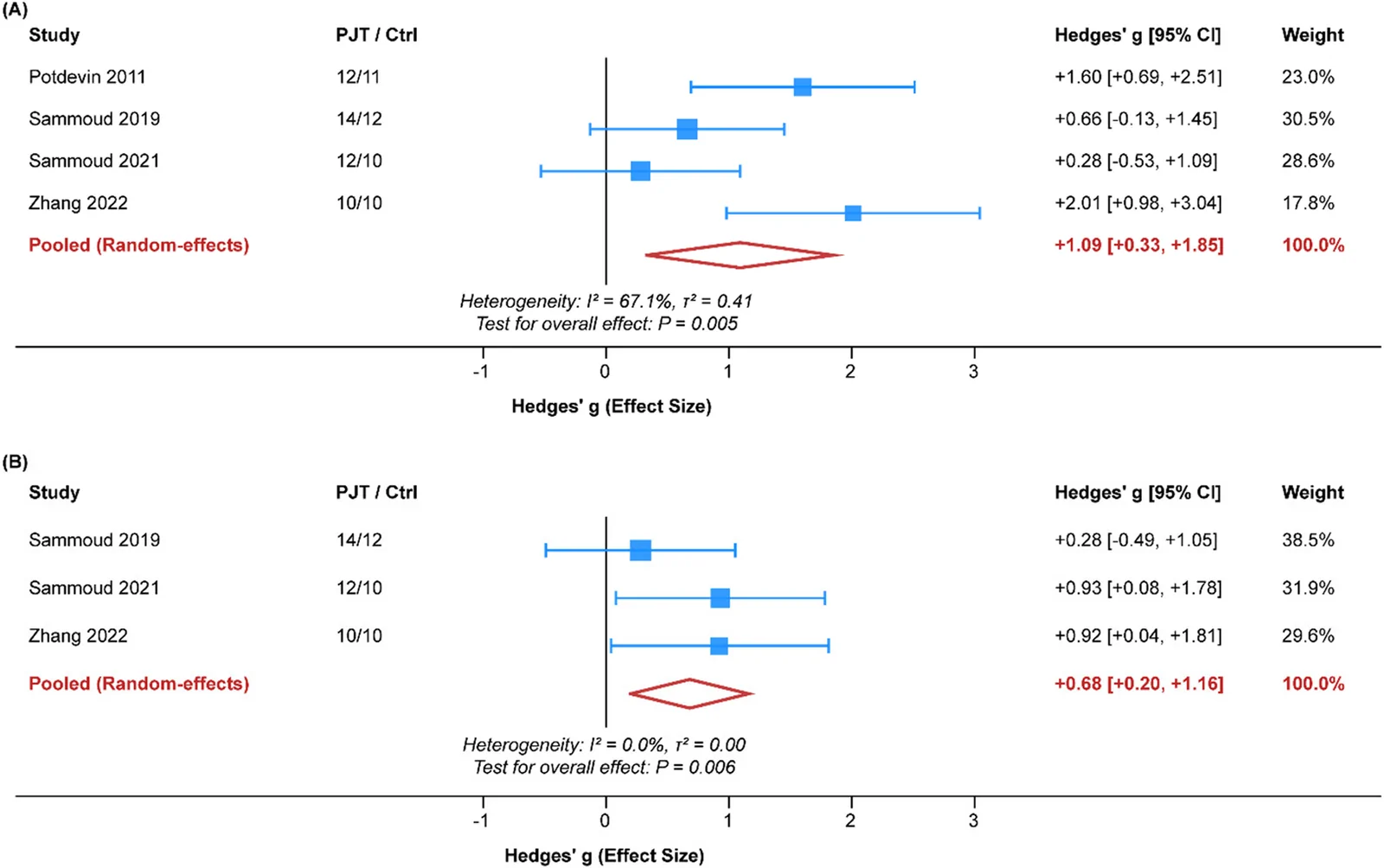
Forest plots of the meta-analysis for explosive power (**A**: CMJ, **B**: SLJ)

Taking the countermovement jump (CMJ) height as the gold-standard indicator of explosive power, Fig. 3(A) presents the random-effects pooled results of 4 studies (*n* = 91), where PT produced a large effect compared with the control condition, with Hedges’ *g* = + 1.09 (95% confidence interval (CI) − 0.17 to + 2.36, *P* = 0.071), and individual effects were + 1.60 in [19], + 0.66 in [12], + 0.28 in [13] and + 2.01 in [22]. The heterogeneity was *I*^2^ = 67.1% (Q = 9.12, *P* = 0.028), indicating a meaningful between-study difference in effect magnitude, and the leave-one-out analysis showed that the pooled *g* remained within + 0.82 to + 1.37 after omitting any single study, which indicates that the location of the central estimate is stable; notably, stability of the point estimate does not itself imply statistical significance, and the pooled interval under so few studies does not exclude the null value. A sensitivity analysis restricted to low-risk studies (k = 3) yielded *g* = + 0.82 (95% CI + 0.08 to + 1.56, *P* = 0.029), with the direction and magnitude of the estimate preserved. This nominally significant value derives from a low-risk subset of three studies and does not alter the non-significant status of the full pooled estimate under the primary Hartung–Knapp analysis.

Figure 3(B) presents the meta-analytic result for the standing long jump (SLJ) as an auxiliary indicator of horizontal explosive power (k = 3, *n* = 68), yielding a moderate point estimate whose interval, under only three studies, does not exclude the null value, with *g* = + 0.68 (95% CI − 0.38 to + 1.73, *P* = 0.110, *I*^*2*^ = 0%), and individual effect sizes of + 0.93 in [13], + 0.92 in [22] and + 0.28 in [12], with all three studies aligned in the same direction.

The CMJ and SLJ pooled results jointly demonstrate that PT can simultaneously improve both vertical and horizontal lower-limb explosive power output, with a consistent gradient of effect across the two dimensions, as further detailed in Table 2.

**Table 2:** Pooled effect sizes and heterogeneity by outcome

Outcome	Category	k	*n* (PJT/Ctrl)	Hedges’ g	95% CI	*P*	I^2^ (%)	τ^2^
CMJ	Explosive power	4	48/43	+ 1.09	[− 0.17, + 2.36]	0.071	67.1	0.42
SLJ	Explosive power	3	36/32	+ 0.68	[− 0.38, + 1.73]	0.110	0.0	0.00
25 m KWP	Swim - mechanism	2	26/22	+ 1.35	descriptive (k = 2)	—	0.0	0.00
25 m WP	Swim - short	2	26/22	+ 0.73	descriptive (k = 2)	—	0.0	0.00
25 m Front Crawl	Swim - short	3	36/32	+ 0.78	[− 0.88, + 2.44]	0.181	55.7	0.24
50 m Front Crawl	Swim - core	5	67/62	+ 0.33	[− 0.16, + 0.82]	0.139	0.0	0.00
15 m Front Crawl	Swim - start	2	25/22	+ 0.33	descriptive (k = 2)	—	1.6	0.00
Start distance	Swim - start	2	21/21	+ 1.32	[− 0.95, + 3.59]	—	91.0	2.44

Pooled estimates use restricted maximum likelihood for the between-study variance with Hartung–Knapp–Sidik–Jonkman confidence intervals; outcomes informed by only two studies are reported descriptively because the Hartung–Knapp interval is not interpretable at that number of studies. The DerSimonian–Laird results are provided as a sensitivity analysis in Table S2

*CI* confidence interval, *g* Hedges’ g, *I*^*2*^  inconsistency index, *k* number of studies, *P* two-sided P value, τ^*2*^  between-study variance

### Meta-analytic findings for swimming performance: 50 m front crawl as the core event anchor

**Fig. 4:**
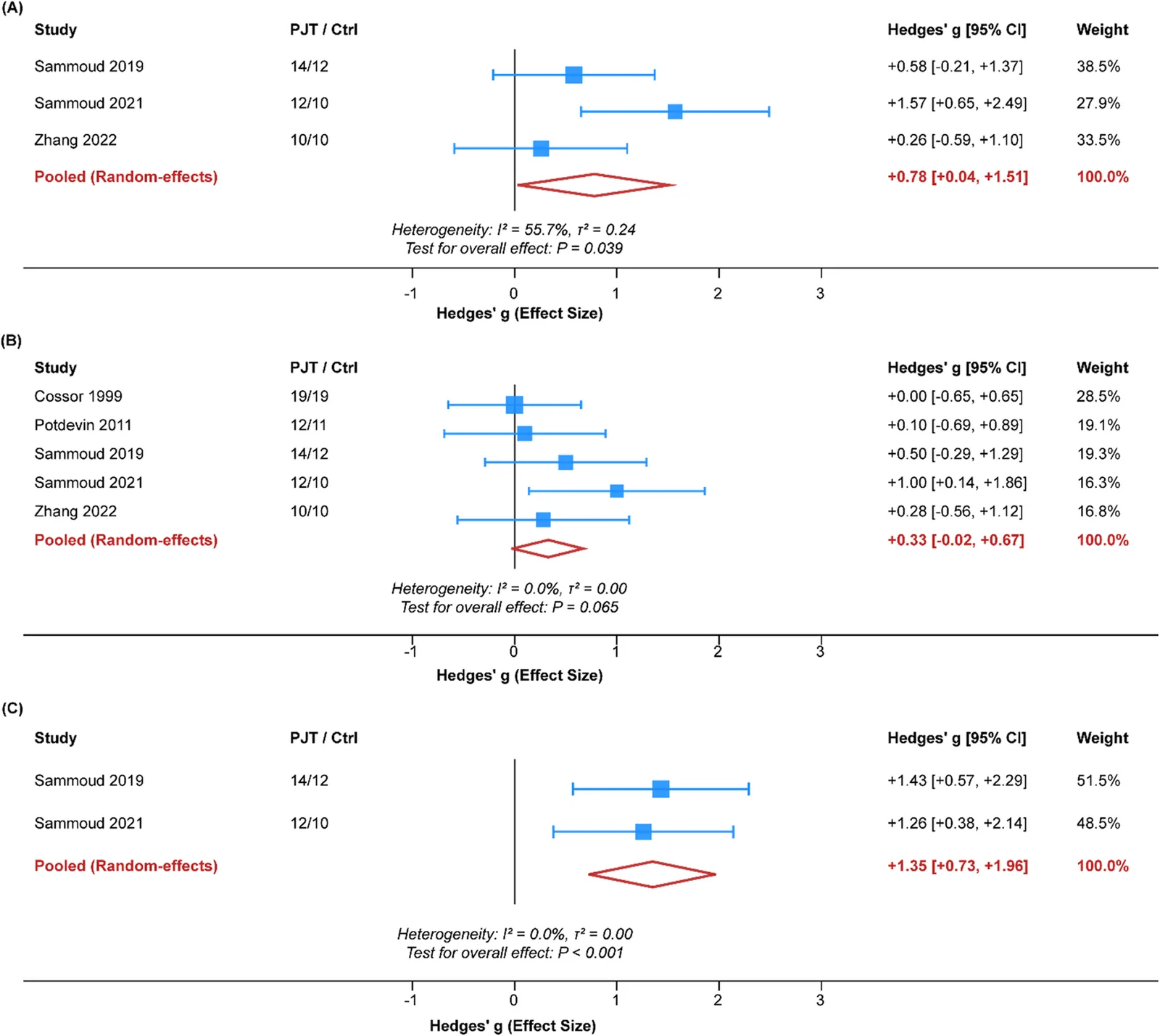
Forest plots of the meta-analysis for swimming performance (**A**: 25 m, **B**: 50 m, **C**: 25 m KWP)

Figure 4(A) presents the meta-analytic result for 25 m front-crawl time (k = 3, *n* = 68), with a pooled effect size of *g* = + 0.78 (95% CI − 0.88 to + 2.44, *P* = 0.181, *I*^2^ = 55.7%), with individual effects of + 0.58 in [12], + 1.57 in [13] and + 0.26 in [22], indicating a moderate-to-large point estimate whose interval, under only three studies, remains wide and does not exclude the null value.

Figure 4(B) presents the meta-analytic result for 50 m front-crawl time as the most representative competitive indicator (k = 5, *n* = 129), with a pooled effect size of *g* = + 0.33 (95% CI − 0.16 to + 0.82, *P* = 0.139, *I*^2^ = 0%), indicating a small point estimate that the pooled interval does not distinguish from the null value, with the 5 studies uniformly showing modest effect magnitudes ([21]*g* = 0.00 [19], *g* = + 0.10 [12], *g* = + 0.50 [13], *g* = + 1.00 and [22]*g* = + 0.28). The leave-one-out analysis showed that after excluding [21] (a 20-week partial-swim replacement protocol), the pooled *g* rose to + 0.45, which indicates that the point estimate is sensitive to this single study and suggests that the null finding of [21] exerted a dilutive influence on the overall effect. The comparison between Fig. 4(A) and Fig. 4(B) shows a distance-related gradient in the point estimates, with a larger central value at 25 m than at 50 m, a pattern consistent with a short-distance advantage of PT that the present sparse evidence base is nonetheless unable to confirm inferentially.

Figure 4(C) presents the result for the 25 m kick-without-push (KWP) test (k = 2, *n* = 48), for which the pooled point estimate was *g* = + 1.35, and because only two studies contributed the Hartung–Knapp interval reduces to a single degree of freedom and is not interpretable, so this outcome is summarised descriptively through its point estimate and the agreement of the contributing studies, because outcomes informed by only two studies cannot provide reliable evidence of effectiveness. With [12] (males, *g* = + 1.43) and [13] (females, *g* = + 1.26) consistent in direction and magnitude. The agreement of these two studies is compatible with a pathway linking lower-limb explosive power to in-water kicking propulsion, although this interpretation rests on only two studies and is best regarded as hypothesis-generating, while auxiliary outcomes at 15 m front-crawl time and 25 m with push exhibited point estimates in the same direction. The pooled estimate for start distance (g = + 1.32, 95% CI − 0.95 to + 3.59, I^2^ = 91.0%) is reported descriptively only and not interpreted inferentially given its null-crossing CI and extreme heterogeneity.

### Linear meta-regression for dose-response relationships

Weighted random-effects meta-regression was applied to evaluate the moderating effects of intervention weeks and total ground contacts on PT effect size.

**Fig. 5:**
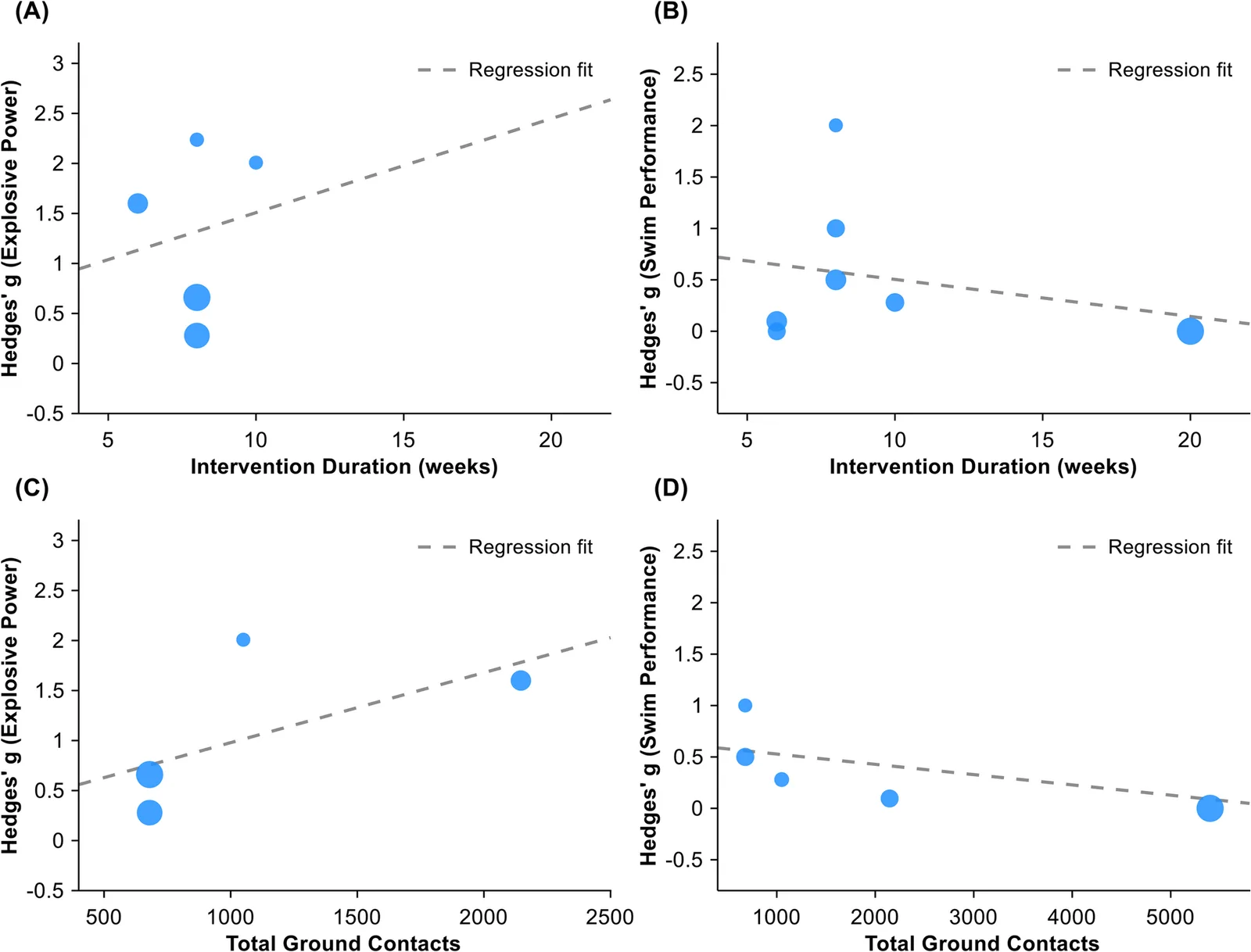
Linear meta-regression of dose-response relationships (**A**: weeks × explosive power, **B**: weeks × swimming, **C**: ground contacts × explosive power, **D**: ground contacts × swimming)

Figure 5(A) presents the relationship between intervention weeks and the explosive-power effect size (k = 5, weeks 6 to 10), with a regression coefficient of *β* = +0.094 per week (95% CI − 0.59 to + 0.78), *P* = 0.786, and *R*^2^ approximately 0, failing to reach statistical significance; given that only five studies entered this model, this coefficient is not reliably interpretable and is reported solely for transparency. Figure 5(B) presents the relationship between intervention weeks and the swimming-performance effect size (k = 7, weeks 6 to 20), with *β* = −0.036 per week (95% CI − 0.15 to + 0.07), *P* = 0.515, likewise non-significant. With only five and seven studies entering these two models, the analyses are severely underpowered for detecting a linear trend, so the non-significant coefficients are uninformative rather than evidence that intervention weeks has no influence on the PT effect. Critically, non-significant *P* values in analyses with so few studies reflect insufficient statistical power and should not be interpreted as demonstrating the absence of a dose-response relationship.

Figure 5(C) presents the relationship between total ground contacts and the explosive-power effect size (k = 4, range 680 to 2146 contacts), with *β* = +0.0007 per contact (95% CI − 0.0004 to + 0.0019), *P* = 0.226, failing to reach significance. Figure 5(D) presents the relationship between total ground contacts and the swimming-performance effect size (k = 5, range 680 to 5400 contacts), with *β* = −0.0001 per contact (95% CI − 0.0003 to 0.0000), *P* = 0.149, likewise non-significant.

None of the four dose-response models detected a significant linear relationship, with regression coefficients non-significant and *R*² values approaching zero, indicating that the pre-specified hypothesis (H2) of a linear positive dose-response gradient could not be reliably tested rather than shown to be absent, because each model rested on only four to seven studies across a narrow dose range and therefore lacked the power that meta-regression requires, for which roughly ten studies per predictor are generally recommended. Inspection of the overall data distribution in Fig. 5 revealed that the three studies with the largest effects [18, 19, 22] all fell within the 6-to-10-week range with total ground contacts no greater than 2146, while the 20-week ultra-long protocol of [21] with 5400 ground contacts and a partial-swim replacement mode produced a null effect. The absence of a testable dose-response function means that no dose threshold can be inferred from these data. Any impression from the scatter that the larger effects clustered at shorter durations and lower contact totals is a post-hoc visual observation only, was not statistically tested, and is reported solely as a hypothesis to be examined in future studies with denser dose coverage and non-linear modelling capacity rather than as a basis for prescribing a particular dose.

### Biological maturation, sex subgroups and robustness checks

This section treats maturation and sex as twin pillars and focuses on the core hypotheses implicit in the study title.

**Fig. 6:**
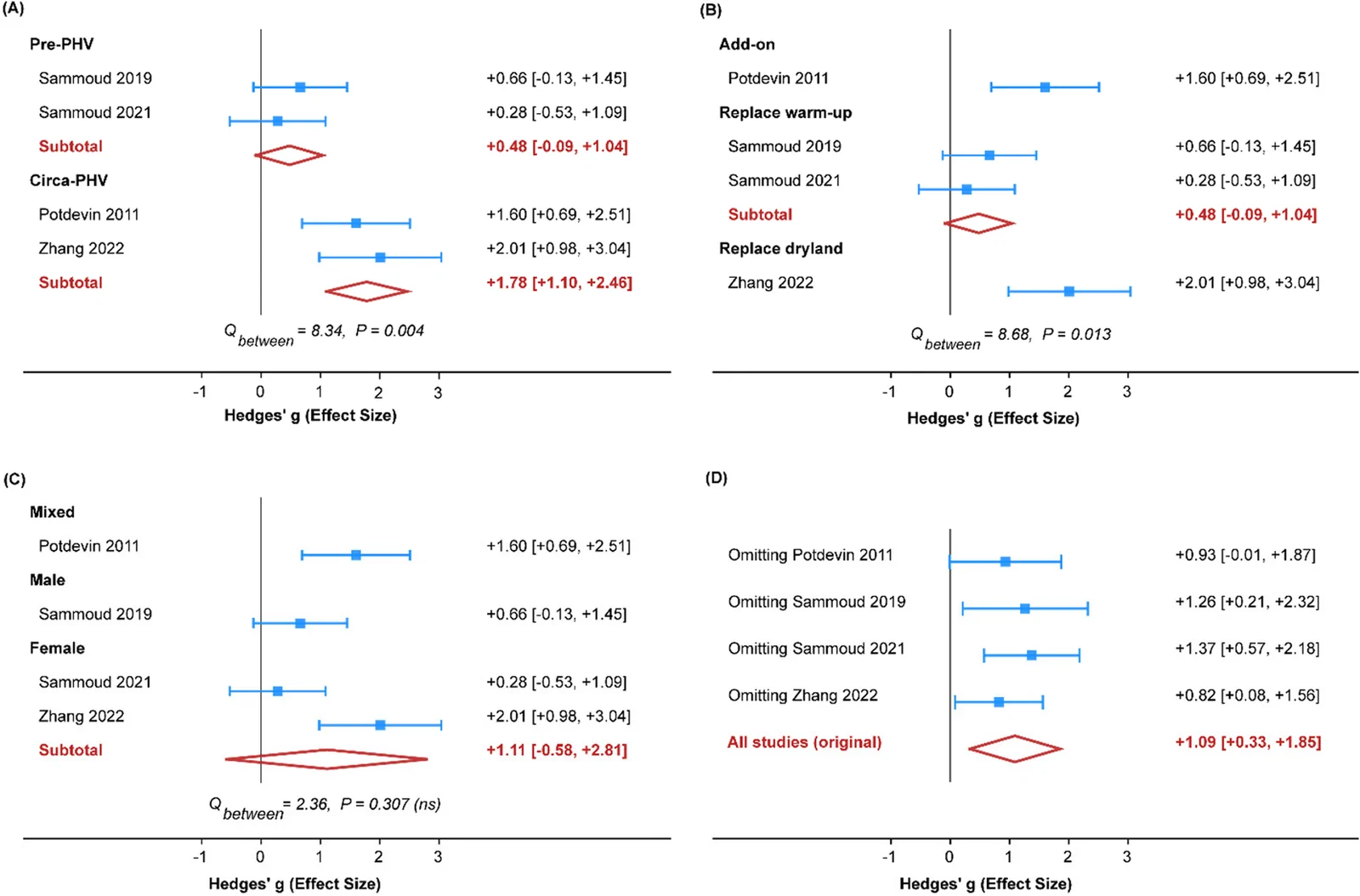
Subgroup and sensitivity analyses (**A**: maturation subgroups, **B**: intervention-mode subgroups, **C**: sex subgroups, **D**: leave-one-out sensitivity)

Figure 6(A) presents the CMJ subgroup meta-analysis stratified by Pre-/Circa-PHV, where the Circa-PHV group [19, 22], each stratum containing only two studies, showed a point estimate of *g* = + 1.78 that was larger than the Pre-PHV group [12, 13] with *g* = + 0.48 (95% CI − 0.09 to + 1.04), Q_*between*_ = 8.34, *P* = 0.004, yet with only two studies in each stratum this between-group contrast is statistically fragile and is presented as an exploratory, hypothesis-generating signal that the Circa-PHV stage may be a more responsive window rather than as confirmatory evidence; with only two studies per stratum, the observed difference could be driven entirely by the characteristics of a single study, and this finding requires replication in larger, adequately powered samples before any conclusion can be drawn, while the Post-PHV group with [20] alone could not be pooled.

Figure 6(B) presents the CMJ subgroup results stratified by intervention mode, where the add-on mode yielded *g* = + 1.60 and the dry-land-replacement mode *g* = + 2.01, both substantially larger than the warm-up-replacement mode with *g* = + 0.48, Q_*between*_ = 8.68, *P* = 0.013, although, as noted below, each mode was represented by very few studies, so this contrast indicates only that the point estimates differed across modes and is compatible with weaker independent stimulus exposure when PT is embedded within the warm-up window. Because the add-on and dry-land-replacement categories each comprised a single study, this modal comparison is exploratory and the two single-study values should not be read as pooled estimates.

Figure 6(C) presents the CMJ results stratified by sex, with *g* = + 1.11 in the female group, *g* = + 0.66 in the male group and *g* = + 1.60 in the mixed-sex group, Q_*between*_ = 2.36, *P* = 0.307, indicating no statistically significant between-sex difference. The mixed-sex category rested on a single study that also carried the highest individual effect, so with very few studies per sex the null between-sex result should be read with caution rather than as equivalence. Any post-training standard deviation issues identified in [13] were handled within the sensitivity analysis framework.

Figure 6(D) presents the leave-one-out sensitivity analysis for the CMJ pooled estimate, which showed that the pooled *g* remained stable within the + 0.82 to + 1.37 range after omitting any single study, which indicates that the location of the central estimate does not depend on any one study, even though the pooled interval under so few studies does not exclude the null value.Formal assessment of publication bias through funnel plot inspection and Egger testing was not performed given that k = 8 fell below the recommended threshold.

Within the GRADE framework, and after removing the large-effect upgrade that the wider Hartung–Knapp intervals no longer justify, no outcome retained high or moderate certainty. CMJ and 25 m front-crawl time were rated as very low certainty, while 25 m KWP, SLJ and 50 m front-crawl time were rated as low certainty, as detailed in Table 3.

**Table 3:** GRADE summary of findings

Outcome	k	*n*	Hedges’ g (95% CI)	Risk of bias	Inconsistency	Imprecision	Large effect	Certainty
CMJ	4	91	+ 1.09 [− 0.17, + 2.36]	Serious (− 1)	Serious (-1)	Serious (− 1)	No	⨁〇〇〇 Very low
25 m KWP	2	48	+ 1.35 descriptive (k = 2)	Not serious	Not serious	Very serious (− 2)	No	⨁⨁〇〇 Low
SLJ	3	68	+ 0.68 [− 0.38, + 1.73]	Serious (− 1)	Not serious	Serious (-1)	No	⨁⨁〇〇 Low
50 m Front Crawl	5	129	+ 0.33 [− 0.16, + 0.82]	Serious (− 1)	Not serious	Serious (-1)	No	⨁⨁〇〇 Low
25 m Front Crawl	3	68	+ 0.78 [− 0.88, + 2.44]	Serious (− 1)	Serious (-1)	Serious (-1)	No	⨁〇〇〇 Very low

-1 = downgraded one level. +1 = upgraded one level. Publication bias was not formally assessed (k < 10). Although the 50 m front crawl 95% CI also crosses zero, it was rated as low rather than very low certainty because the difference in overall certainty stems from the inconsistency domain, not from a different imprecision rating. All three outcomes received the same Serious (− 1) downgrade for imprecision; however, CMJ and 25 m front crawl were additionally downgraded for statistical inconsistency (CMJ I^2^ = 67.1%; 25 m front crawl I^2^ = 55.7%), whereas 50 m front crawl showed no inconsistency (I² = 0%) and therefore received no such downgrade. It should also be noted that the reviewer’s premise of similarly wide confidence intervals does not hold: the 50 m front crawl CI spans 0.98 units, which is substantially narrower than the CMJ interval (2.53 units) and the 25 m front crawl interval (3.32 units)

*GRADE* Grading of Recommendations, Assessment, Development and Evaluation

## Discussion

The spectrum of PT effects presented in the present study shows point estimates that are larger at the explosive-power end and smaller as swimming distance increases, and although the sparse evidence base means this gradient cannot be confirmed inferentially, its direction is consistent with a biomechanical selectivity in the transfer from explosive power to sport-specific performance. Lower-limb explosive-power pooled effects previously reported in mixed-sport adolescent populations have generally clustered at moderate magnitudes [23], and a recent controlled trial that integrated plyometric training into physical-education sessions over four weeks in older adolescents likewise reported a large countermovement-jump effect [24]. Comparing the two contexts is informative: the school-based cohort in [24] comprised older adolescents drawn from general physical-education classes who were already habituated to a variety of land-based activities, whereas the swimmers in the present synthesis were competitive athletes whose training is dominated by a weight-supported aquatic environment with limited exposure to high-impact dry-land loading. Despite its shorter duration (four weeks versus the six-to-ten-week range of most swimming studies), the school-based trial still produced a large CMJ improvement, yet the swimming-specific pooled point estimate (g = + 1.09) appeared even larger. This contextual contrast suggests that the magnitude of the plyometric response may be modulated by prior training history: swimmers, who lack habitual ground-reaction-force stimuli, may possess a relatively larger untapped adaptive window for explosive-power gains than school students who already perform regular land-based physical activity, and this disparity suggests that aquatic athletes who operate in a weight-supported environment have long experienced a training gap in high-impact dry-land stimuli, thereby leaving a relatively larger adaptive window and a steeper marginal benefit curve for PT. At the same time, while reviews of strength training for short- and middle-distance swimming have revealed the decisive role of strength methodology for front-crawl performance [25], and conventional strength periodisation interventions in adolescent swimmers have failed to produce significant effects on swimming time [26], the present synthesis adds a maturation-stratified estimate of the isolated PT effect, although the width of the pooled intervals means the dose boundaries remain provisional rather than firmly established. Even more noteworthy is that the cross-sport pooled effect reported in the most recent adolescent complex-PT review [27] assumes a clearer window specificity once stratified by developmental stage as in the present synthesis, thereby offering an incremental, though still preliminary, contribution along the three fronts of adolescent-population specificity, dose-effect description and event-distance gradient.

Because none of the included studies directly measured mechanistic variables and the evidence certainty is very low to low, the following mechanistic interpretation is hypothetical and draws on indirect evidence from the wider literature rather than on data from the present synthesis. The performance improvements driven by PT cannot be explained by any single pathway, and the underlying mechanisms require coupled interpretation across three tiers comprising neuromuscular adaptation, the force-velocity curve and dry-land-to-water transfer. At the neuromuscular tier, the adaptations manifest as reductions in motor-unit recruitment threshold, elevations in musculotendinous stiffness and improvements in SSC energy-utilisation efficiency, and the differential response gradient across adolescent groups of varying biological maturation indicates that the trainability of SSC efficiency itself is deeply modulated by developmental status [28], which offers a plausible neurophysiological reading of the larger point estimate seen in the circa-PHV stratum, a stratum that nonetheless comprised only two studies and is therefore exploratory. At the force-velocity-curve tier, the ballistic-contraction stimulus shifts the high-velocity end of the curve rightwards and selectively enhances force output under short-duration and high-contraction-velocity conditions, and this mechanism aligns closely with the interaction patterns between kinematic, kinetic and energetic predictors of young swimmers’ speed [29], implying that what PT reshapes is not the absolute ceiling of maximal strength but the capacity to release already accumulated strength efficiently within a short time window. At the dry-land-to-water transfer tier, the robust association between force production in elite swimmers and inter-lap pacing together with stroke kinematics in 100 m front crawl suggests that the mapping from dry-land explosive power to in-water propulsion favours short-duration impulsive actions as the priority channel [30], which mechanistically accounts for the finding that non-cyclic high-power phases such as the start, turn and 25 m KWP carried the larger point estimates in the present study, an observation that remains exploratory given the small number of studies; critically, these larger point estimates each rest on only one or two studies, so they are preliminary and should not be interpreted as evidence of effectiveness until replicated in adequately powered trials. More targeted still, a single comparative trial in adolescent swimmers has suggested that vertical-jump training offers a distinctive short-duration power advantage over maximal-strength training for start performance [20], and this additional evidence reinforces the close match between the short-duration high-power output strengthened by PT and the mechanical signature of the non-cyclic technical phases in swimming, while the weak coupling of PT to the steady-state energy-metabolism profile of cyclical stroking precisely constitutes the mechanistic basis of the diminishing effect at 50 m and beyond.

Informed by the distribution pattern across studies rather than by a statistically supported dose-response function, the included studies that produced the largest effects happened to cluster within ranges of roughly 8 to 10 weeks, 2 to 3 sessions per week and 50 to 120 ground contacts per session with progressive overload; however, because the meta-regression detected no significant dose-response relationship, this clustering is a purely descriptive, post-hoc observation that carries no statistical basis and should not be interpreted as an evidence-based dose recommendation. These observed parameter ranges nonetheless overlap with the sensitive range identified in the most recent adolescent soccer PT synthesis [25], and it resonates with the cross-sport ceilings of 400 to 600 min of total intervention time and 900 to 1400 total ground contacts emphasised in wider meta-analytic work [23], and this cross-sport consistency indicates that the pedagogical principle that prolonged periodisation and excessive contact accumulation yield no additional benefit and may instead reach an adaptation plateau holds general applicability within the adolescent population. Individualised prescription grounded in biological maturation should adhere to technical learning and low-impact jumping as the focus of the Pre-PHV stage, may treat the circa-PHV stage as a potentially more responsive window within which moderate progression could be considered, and should reconsider training strategies in the Post-PHV stage where the dual attenuation of central and peripheral responsiveness becomes relevant, and the layered logic of this developmental window has been consistently supported in sprint adaptation research on male team-sport youth [31], providing a solid evidence base for coaches to adopt biological maturation rather than chronological age as the benchmark for prescription adjustment. Programme implementation should be embedded within the annualised training cycle in parallel with landing-technique education, training-load monitoring and injury-prevention principles, and the international consensus on youth resistance and power training emphasises that professional supervision and individualised progression are the preconditions for reconciling safety and efficacy [32], which further consolidates from the governance-of-practice perspective the positioning of PT as a functional supplement rather than a replacement for in-water training in adolescent swimmers.

Several methodological limitations should be acknowledged. Each outcome rested on only a few studies, so the pooled Hartung–Knapp intervals were wide and did not exclude the null value, which makes the estimates preliminary rather than confirmatory. Maturation was in most studies inferred from chronological age rather than measured through maturity offset or Tanner staging, so the apparently greater circa-PHV responsiveness rests on an imprecise classification. The post-PHV stage was represented by a single trial that used an active maximal-strength-training comparator, which limits inference about effect attenuation across the developmental window. The 25 m kick-without-push result derived from only two trials by the same research group in pre-PHV samples, so its generalisability to other stages and populations is uncertain. The dose-response analysis could examine only intervention duration and total ground contacts; training intensity and progressive overload were reported too heterogeneously across studies to be quantified, so their dose-response relationships could not be examined, which is a further limitation.The pooled control conditions were heterogeneous, so the between-mode differences and the dilutive influence of the single 20-week protocol may reflect training volume rather than the plyometric stimulus itself. Publication bias could not be tested formally with fewer than ten studies, and although the search extended to CNKI, grey literature and citation tracking, a modest over-estimation of the effect cannot be excluded. The extreme heterogeneity of the start-distance estimate reflected marked differences in risk of bias, sample and protocol between its two contributing trials, so that outcome was reported descriptively only.Future research should undertake maturation-stratified, sex-balanced and standardised randomised controlled trials that couple dry-land kinetic measurement with three-dimensional in-water kinematic analysis to characterise the transfer pathway.

## Supplementary Information

Supplementary Material 1. Table S1

Supplementary Material 2. Table S2

## Data Availability

All data generated or analysed during this study are included in this published article and its supplementary information files.

## Abbreviations

CI: Confidence interval
CMJ: Countermovement jump
CNKI: China National Knowledge Infrastructure
FC: Front crawl
GRADE: Grading of Recommendations, Assessment, Development and Evaluation
KWP: Kick without push
MST: Maximal strength training
PEDro: Physiotherapy Evidence Database
PHV: Peak height velocity
PICOS: Population, Intervention, Comparator, Outcome and Study design
PJT: Plyometric jump training
PRISMA: Preferred Reporting Items for Systematic Reviews and Meta-Analyses
PT: Plyometric training
RCT: Randomised controlled trial
RoB 2.0: Cochrane Risk of Bias tool version 2.0
SJ: Squat jump
SLJ: Standing long jump
SSC: Stretch-shortening cycle
WP: With push

## References

[1] Ruiz-Navarro JJ, Santos CC, Born D-P, López-Belmonte Ó, Cuenca-Fernández F, Sanders RH, et al. Factors relating to sprint swimming performance: a systematic review. Sports Med. 2025;55:899–922. 10.1007/s40279-024-02172-4. 39841367 PMC12011652

[2] Carvalho DD, Monteiro AS, Fonseca P, Silva AJ, Vilas-Boas JP, Pyne DB, et al. Swimming sprint performance depends on upper/lower limbs strength and swimmers level. J Sports Sci. 2023;41:747–57. 10.1080/02640414.2023.2239610. 37488696

[3] Keiner M, Wirth K, Fuhrmann S, Kunz M, Hartmann H, Haff GG. The influence of upper- and lower-body maximum strength on swim block start, turn, and overall swim performance in sprint swimming. J Strength Cond Res. 2021;35:2839–45. 10.1519/JSC.0000000000003229. 31425457

[4] Retzepis N-O, Avloniti A, Kokkotis C, Stampoulis T, Balampanos D, Gkachtsou A, et al. The effect of peak height velocity on strength and power development of young athletes: a scoping review. J Funct Morphol Kinesiol. 2025;10:168. 10.3390/jfmk10020168. 40407452 PMC12101259

[5] Faigenbaum AD, Kraemer WJ, Blimkie CJ, Jeffreys I, Micheli LJ, Nitka M, et al. Youth resistance training: updated position statement paper from the national strength and conditioning association. J Strength Cond Res. 2009;23:S60–79. 10.1519/JSC.0b013e31819df407. 19620931

[6] Radnor JM, Oliver JL, Waugh CM, Myer GD, Moore IS, Lloyd RS. The influence of growth and maturation on stretch-shortening cycle function in youth. Sports Med. 2018;48:57–71. 10.1007/s40279-017-0785-0. 28900862 PMC5752749

[7] Lloyd RS, Oliver JL, Faigenbaum AD, Howard R, De Ste Croix MBA, Williams CA, et al. Long-term athletic development- part 1: a pathway for all youth. J Strength Cond Res. 2015;29:1439–50. 10.1519/JSC.0000000000000756. 25486295

[8] Ramirez-Campillo R, García-Hermoso A, Moran J, Chaabene H, Negra Y, Scanlan AT. The effects of plyometric jump training on physical fitness attributes in basketball players: A meta-analysis. J Sport Health Sci. 2022;11:656–70. 10.1016/j.jshs.2020.12.005. 33359798 PMC9729929

[9] Crowley E, Harrison AJ, Lyons M. The Impact of Resistance Training on Swimming Performance: A Systematic Review. Sports Med. 2017;47:2285–307. 10.1007/s40279-017-0730-2. 28497283

[10] Fone L, Van Den Tillaar R. Effect of different types of strength training on swimming performance in competitive swimmers: A systematic review. Sports Med - Open. 2022;8:19. 10.1186/s40798-022-00410-5. 35099631 PMC8804114

[11] Thng S, Pearson S, Keogh JW. Relationships between dry-land resistance training and swim start performance and effects of such training on the swim start: a systematic review. Sports Med. 2019;49:1957–73. 10.1007/s40279-019-01174-x. 31493205

[12] Sammoud S, Negra Y, Chaabene H, Bouguezzi R, Moran J, Granacher U. The effects of plyometric jump training on jumping and swimming performances in prepubertal male swimmers. J Sports Sci Med. 2019;18:805. 31827366 PMC6873130

[13] Sammoud S, Negra Y, Bouguezzi R, Hachana Y, Granacher U, Chaabene H. The effects of plyometric jump training on jump and sport-specific performances in prepubertal female swimmers. J Exerc Sci Fit. 2021;19:25–31. 10.1016/j.jesf.2020.07.003. 32922460 PMC7475125

[14] Page MJ, McKenzie JE, Bossuyt PM, Boutron I, Hoffmann TC, Mulrow CD, et al. The PRISMA 2020 statement: an updated guideline for reporting systematic reviews. BMJ. 2021;372. 10.1136/bmj.n71. PMC8005924 33782057

[15] Sterne JA, Savović J, Page MJ, Elbers RG, Blencowe NS, Boutron I, et al. RoB 2: a revised tool for assessing risk of bias in randomised trials. BMJ. 2019;366. 10.1136/bmj.l4898. 31462531

[16] Guyatt GH, Oxman AD, Vist GE, Kunz R, Falck-Ytter Y, Alonso-Coello P, et al. GRADE: an emerging consensus on rating quality of evidence and strength of recommendations. BMJ. 2008;336:924–6. 10.1136/bmj.39489.470347.AD. 18436948 PMC2335261

[17] Tartaleanu C, Ortanescu D. Improving the start in swimmers with the help of plyometric exercises. Bull Transilv Univ Braş IX: Sci Hum Kinet. 2024;:17–24.

[18] Bishop D, Smith R, Smith M, Rigby H. Effect of plyometric training on swimming block start performance in adolescents. J Strength Cond Res (Natl Strength Cond Assoc). 2009;23:2137–43. 10.1519/JSC.0b013e3181b866d0. 19855343

[19] Potdevin FJ, Alberty ME, Chevutschi A, Pelayo P, Sidney MC. Effects of a 6-week plyometric training program on performances in pubescent swimmers. J Strength Cond Res. 2011;25:80–6. 10.1519/JSC.0b013e3181fef720. 21157388

[20] Born D-P, Stöggl T, Petrov A, Burkhardt D, Lüthy F, Romann M. Analysis of freestyle swimming sprint start performance after maximal strength or vertical jump training in competitive female and male junior swimmers. J Strength Conditioning Res. 2020;34:323–31. 10.1519/JSC.0000000000003390. 31985714

[21] Cossor J, Blanksby B, Elliott B. The influence of plyometric training on the freestyle tumble turn. J Sci Med Sport. 1999;2:106–16. 10.1016/s1440-2440(99)80190-x. 10476974

[22] Zhang Y. Kuaisu shensuo fuhe xunlian dui duanjuli youyong yundong biaoxian de yingxiang yanjiu [Research on the effect of plyometric training on short-distance swimming performance: taking 12–14-year-old female swimmers as an example] [master’s thesis]. Shijiazhuang: Hebei Normal University; 2022. 10.27110/d.cnki.ghsfu.2022.001408. Chinese.

[23] Chen L, Zhang Z, Huang Z, Yang Q, Gao C, Ji H, et al. Meta-analysis of the effects of plyometric training on lower limb explosive strength in adolescent athletes. Int J Environ Res Public Health. 2023;20:1849. 10.3390/ijerph20031849. 36767213 PMC9915200

[24] Alonso-Aubin DA, Saez-Berlanga Á, Chulvi-Medrano I, Martínez-Guardado I. Effects of integrating a plyometric training program during physical education classes on ballistic neuromuscular performance. J Funct Morphol Kinesiol. 2025;10:240. 10.3390/jfmk10030240. 40700176 PMC12286031

[25] Jin G, Jin Y, Zhang H, Fu X, Yang Y, Lin S-C. The methodology of resistance training is crucial for improving short-medium distance front crawl performance in competitive swimmers: A systematic review and meta-analysis. Front Physiol. 2024;15:1406518. 10.3389/fphys.2024.1406518. 39129754 PMC11310150

[26] Schumann M, Notbohm H, Bäcker S, Klocke J, Fuhrmann S, Clephas C. Strength-training periodization: No effect on swimming performance in well-trained adolescent swimmers. Int J Sports Physiol Perform. 2020;15:1272–80. 10.1123/ijspp.2019-0715. 32126524

[27] Zhang J, Wu X, Ye X. The effect of plyometric complex training on lower-limb explosive power in adolescents: a systematic review and meta-analysis. Front Physiol. 2025;16:1716568. 10.3389/fphys.2025.1716568. 41415306 PMC12708270

[28] Mavili C, Celik H, Unver E, Zengin HY, Cinemre SA. Investigation of the stretch-shortening cycle performance of youth female volleyball players according to different biological maturation and training status. Percept Mot Skills. 2025;00315125251358284. 10.1177/00315125251358284. 40621625

[29] Morais JE, Barbosa TM, Bragada JA, Nevill AM, Marinho DA. Race analysis and determination of stroke frequency–stroke length combinations during the 50-M freestyle event. J Sports Sci Med. 2023;22:156. 10.52082/jssm.2023.156. 36876182 PMC9982526

[30] Costa MJ, Santos CC, Ferreira F, Arellano R, Vilas-Boas JP, Fernandes RJ. Association between elite swimmers’ force production and 100 m front crawl inter-lap pacing and kinematics. Front Sports Act Living. 2023;5:1205800. 10.3389/fspor.2023.1205800. 37305663 PMC10250633

[31] Mendonça FR, de Faria WF, da Silva JM, Massuto RB, Dos Santos GC, Correa RC, et al. Effects of aerobic exercise combined with resistance training on health-related physical fitness in adolescents: a randomized controlled trial. J Exerc Sci Fit. 2022;20:182–9. 10.1016/j.jesf.2022.03.002. 35401769 PMC8958256

[32] Lloyd RS, Faigenbaum AD, Stone MH, Oliver JL, Jeffreys I, Moody JA, et al. Position statement on youth resistance training: the 2014 international consensus. Br J Sports Med. 2014;48:498–505. 10.1136/bjsports-2013-092952. 24055781

