# Supplementary material for: Effects of plyometric training on explosive power and swimming performance in adolescent swimmers: a systematic review and meta-analysis with dose-response analysis
Source: BMC Sports Sci Med Rehabil. 2026 Aug 14;18:407. doi: 10.1186/s13102-026-01989-y (PMC13555939; doi:10.1186/s13102-026-01989-y)
Supplement: Supplementary file 1 [file 13102_2026_1989_MOESM1_ESM.docx]

**Table S1. Records sought but not retrieved, and records excluded at title-and-abstract screening**

**Section A. Reports sought for full-text retrieval but not retrieved within the search window (n = 3).**

| First author / year | Title | Source | Reason not retrieved |
| --- | --- | --- | --- |
| Cossor, J. M. et al. 1999 | The influence of plyometric training on the freestyle tumble turn | Journal of Science and Medicine in Sport 2(2):106–116 | Closed-access (Elsevier); subscription required. Inter-library document delivery request placed but not received within the search window. (Subsequently obtained from the corresponding author via ResearchGate.) |
| Born, D.-P. et al. 2020 | Analysis of freestyle swimming sprint start performance after maximal strength or vertical jump training | Journal of Strength & Conditioning Research 34(2):323–331 | Closed-access (LWW/Wolters Kluwer); subscription required. ILL request placed but not received within the search window. (Subsequently obtained directly from the corresponding author.) |
| Tartaleanu, C. & Ortanescu, D. 2024 | Improving the start in swimmers with the help of plyometric exercises | Bulletin of the Transilvania University of Brasov, Series IX 17(2):17–24 | Regional Romanian journal not indexed in major full-text aggregators; full text obtained from the journal's institutional repository after extended retrieval window. |

*Note. All three reports were subsequently obtained and included in the final synthesis. They are listed here for transparency about the PRISMA-2020 'reports sought / not retrieved within search window' step.*

**Section B. Records excluded at title-and-abstract screening (n = 84), by reason category.**

| Reason category (code) | n | Description |
| --- | --- | --- |
| E1 — Age outside 10–18 y | 11 | Mean sample age <10 or >18 years, or sample described as adult/elite competitive swimmers without an adolescent subgroup. |
| E2 — Non-swimmer athletes | 4 | Sample comprised athletes from other sports (e.g., football, basketball, water polo) or non-athletes. |
| E3 — Special populations | 5 | Disabled swimmers, post-injury rehabilitation cohorts, older-adult populations. |
| E4 — Outcomes not relevant | 4 | Bone density, body composition, shoulder proprioception, biomarker outcomes only. |
| E6 — Acute / post-activation effect | 6 | Single-session PAP / PAPE designs without a chronic training phase ≥ 4 weeks. |
| E9 — No control group | 3 | Single-arm pre–post designs without a control or comparator group. |
| E15 — Review / narrative / meta-analysis | 22 | Systematic reviews, narrative reviews and meta-analyses (cross-checked for primary studies). |
| E16 — Cross-sectional / observational design | 10 | Descriptive, observational or correlational designs without an intervention. |
| E17 — Conference abstract / protocol / unpublished | 15 | Conference proceedings without a full peer-reviewed paper, registered protocols on trial registries, or unpublished theses. |
| E22 — Language | 2 | Reports published in languages other than English or Chinese (Arabic). |
| Other / miscellaneous | 2 | Records that did not fit any of the above categories (e.g., editorial comments, errata). |
| Total excluded at title/abstract | 84 | — |

*Note. The full per-record screening log (n = 95 records that entered title/abstract screening, with the inclusion/exclusion decision and reason for each record) is available on request from the corresponding author or in the project's open-data archive.*
