# Supplementary material for: Effects of plyometric training on explosive power and swimming performance in adolescent swimmers: a systematic review and meta-analysis with dose-response analysis
Source: BMC Sports Sci Med Rehabil. 2026 Aug 14;18:407. doi: 10.1186/s13102-026-01989-y (PMC13555939; doi:10.1186/s13102-026-01989-y)
Supplement: Supplementary file 2 [file 13102_2026_1989_MOESM2_ESM.docx]

**Table S2. DerSimonian–Laird vs REML + Hartung–Knapp–Sidik–Jonkman sensitivity analysis**

| Outcome | k | DL g [95% CI] (*P*) | REML + HKSJ g [95% CI] (*P*) | Δ g (point) | CI width: DL vs HKSJ | τ^2^ | I^2^ (%) |
| --- | --- | --- | --- | --- | --- | --- | --- |
| CMJ | 4 | +1.09 [+0.33, +1.86] (0.005) | +1.09 [−0.17, +2.36] (0.071) | +0.002 | 1.53 vs 2.53 (+66%) | 0.421 | 67.1 |
| SLJ | 3 | +0.68 [+0.20, +1.16] (0.006) | +0.68 [−0.38, +1.73] (0.110) | −0.001 | 0.96 vs 2.11 (+120%) | 0.000 | 0.0 |
| 25 m FC | 3 | +0.78 [+0.04, +1.51] (0.039) | +0.78 [−0.88, +2.44] (0.181) | +0.002 | 1.47 vs 3.32 (+126%) | 0.240 | 55.6 |
| 50 m FC | 5 | +0.33 [−0.02, +0.67] (0.065) | +0.33 [−0.16, +0.82] (0.139) | +0.000 | 0.69 vs 0.98 (+42%) | 0.000 | 0.0 |
| 25 m KWP | 2 | +1.35 [+0.73, +1.96] (<0.001) | +1.35 [−2.65, +5.34] (0.146) | +0.000 | 1.23 vs 7.99 (+549%) | 0.000 | 0.0 |

*Note. DL, DerSimonian–Laird random-effects with Wald (z-based) intervals. REML + HKSJ, Restricted Maximum Likelihood τ^2^ estimation with the Hartung–Knapp–Sidik–Jonkman adjustment using the modified Knapp–Hartung max(q, 1) variance correction and Student's t (k − 1 df) intervals (IntHout et al., 2014; Röver et al., 2015; Veroniki et al., 2016). CI = confidence interval. CMJ, countermovement jump; SLJ, standing long jump; FC, front crawl; KWP, kick without push. Point estimates differ from DL by ≤ 0.005 across all five outcomes; CI widening under HKSJ ranges from +42% to +549% and is driven by (i) the t-based reference distribution (df = k − 1) and (ii) the modified Knapp–Hartung q-multiplier. REML + HKSJ is the pre-specified primary inferential framework of the review; DL results are reported here for completeness and to allow comparison with earlier swimming-related meta-analyses.*
